# Functional Zonation of the Adult Mammalian Adrenal Cortex

**DOI:** 10.3389/fnins.2016.00238

**Published:** 2016-06-15

**Authors:** Gavin P. Vinson

**Affiliations:** School of Biological and Chemical Sciences, Queen Mary University of LondonLondon, UK

**Keywords:** glomerulosa, fasciculata, reticularis, X-Zone, cell proliferation, cell migration, cortisol, aldosterone

## Abstract

The standard model of adrenocortical zonation holds that the three main zones, glomerulosa, fasciculata, and reticularis each have a distinct function, producing mineralocorticoids (in fact just aldosterone), glucocorticoids, and androgens respectively. Moreover, each zone has its specific mechanism of regulation, though ACTH has actions throughout. Finally, the cells of the cortex originate from a stem cell population in the outer cortex or capsule, and migrate centripetally, changing their phenotype as they progress through the zones. Recent progress in understanding the development of the gland and the distribution of steroidogenic enzymes, trophic hormone receptors, and other factors suggests that this model needs refinement. Firstly, proliferation can take place throughout the gland, and although the stem cells are certainly located in the periphery, zonal replenishment can take place within zones. Perhaps more importantly, neither the distribution of enzymes nor receptors suggest that the individual zones are necessarily autonomous in their production of steroid. This is particularly true of the glomerulosa, which does not seem to have the full suite of enzymes required for aldosterone biosynthesis. Nor, in the rat anyway, does it express MC2R to account for the response of aldosterone to ACTH. It is known that in development, recruitment of stem cells is stimulated by signals from within the glomerulosa. Furthermore, throughout the cortex local regulatory factors, including cytokines, catecholamines and the tissue renin-angiotensin system, modify and refine the effects of the systemic trophic factors. In these and other ways it more and more appears that the functions of the gland should be viewed as an integrated whole, greater than the sum of its component parts.

## Introduction—the standard model

The nature and significance of the zonation of the mammalian adrenal cortex has attracted considerable interest during the fifteen decades following the first description of its three main zones, zona glomerulosa, zona fasciculata, and zona reticularis, by Arnold (1866). Later, Gottschau (1883) was the first to suggest that adrenocortical cells originate in the outer part of the gland, and migrate centripetally, thus becoming phenotypically glomerulosa, then fasciculata and reticularis in sequence. Zwemer et al. (1938) illustrated a direct lineage of cortical cells from the connective tissue capsule. Before the functions of the gland were understood, studies were directed to further descriptions of adrenocortical morphology and development, in mammals and other vertebrates (Dostoiewsky, 1886; Rabl, 1891; Wiesel, 1902). By the 1940s it was clear that the adrenal cortex, as well as the medulla, is crucially involved in the response to stress, and further, that secretions of the cortex fell into three functional classes: androgens, and what Selye and Jensen were the first to call mineralocorticoids and glucocorticoids (Selye, 1946; Selye and Jensen, 1946). Coupled with the observable changes in appearance and abundance of the zones in different physiological situations it then became apparent that the different zones had different functions, and androgens, mineralocorticoids, and glucocorticoids were now thought to be products of the zona reticularis, zona glomerulosa, and zona fasciculata respectively (Vines, 1938; Swann, 1940; Chester Jones, 1957; Deane, 1962). Moreover, their regulation was different, and though the inner adrenocortical zones, fasciculata and reticularis, were dependent on an intact pituitary and the secretion of ACTH, the glomerulosa was not (Chester Jones, 1957; Deane, 1962; Vinson, 2003). Later, the essential role of the renin-angiotensin system in the regulation of aldosterone was elucidated (Gross, 1958; Laragh et al., 1960; Carpenter et al., 1961), though other factors, possibly many, are also involved (Vinson et al., 1992b; Ehrhart-Bornstein et al., 1998; Mulrow, 1999; Vinson, 2003).

Together, such evidence has led to the development of what may be thought of as the Standard Model of adrenocortical zonation:

- Adrenocortical cells arise in the outer part of the gland and migrate centripetally, with changes in phenotype as they progress from the glomerulosa through the fasciculata to the reticularis, where they undergo apoptosis.
- Each of the three main differentiated zones secretes a specific profile of steroid hormones.
- Each of the zones is under separate and independent regulatory control.

There have, however, always been gaps in interpretation that have scarcely been addressed. Among these is the challenge of unraveling the mechanisms underlying the profound changes of phenotype that the cells undergo during their lifespan. Again, but less frequently considered, what drives cell migration in the mature gland? Additionally, and rarely addressed: what is the significance of the zonal arrangement—what advantage is conferred by the configuration of the different zones as concentric shells?

It is the purpose of this review to examine both whether the Standard Model remains compatible with the literature of the last few years, and also whether there are now clues to these additional problems.

## What do we mean by adrenocortical zonation?

**Figure 1:**
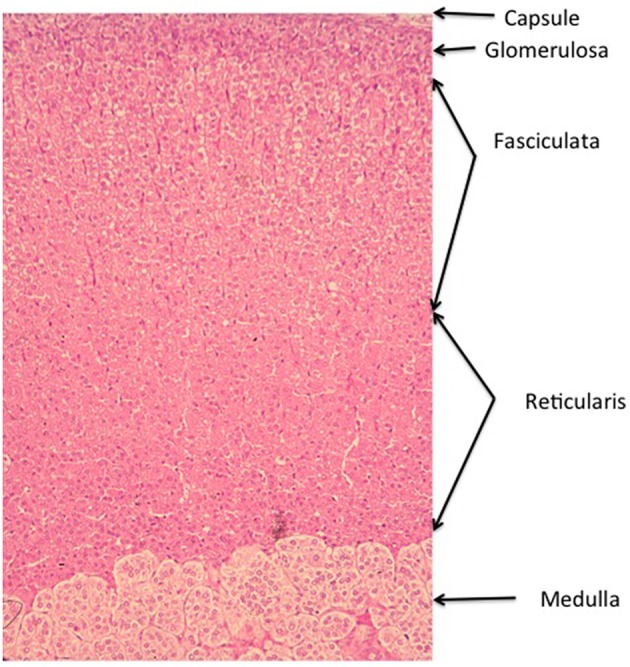
**Zonation of the Wistar rat adrenal cortex**. The main zones of the cortex. The arrangement of the concentric bands of cells is representative of most mammal species, though there is some species variation. In the adult human gland, the glomerulosa is sparse and confined to discrete islets, whereas in others, such as the dog, the glomerulosa is much more marked, with large whorls of cells justifying the comparison with the renal glomerulus. Additional zones may occur in other species, including the X zone in the mouse gland, and the fetal zone in the developing and newborn human gland (See Chester Jones, 1957; Deane, 1962; Neville and O'Hare, 1981; Vinson et al., 1992b).

**Figure 2:**
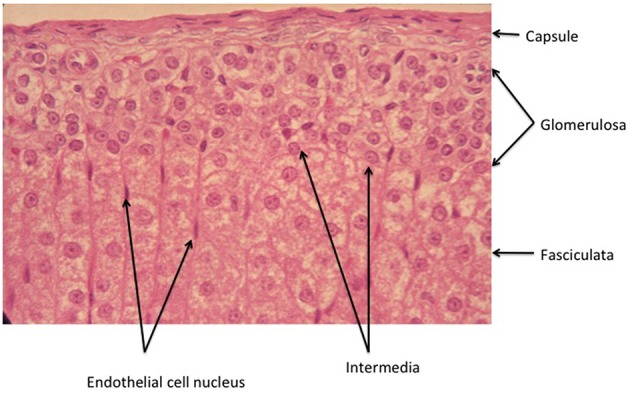
**Higher power view of the capsule/glomerulosa/outer fasciculata view of the rat adrenal cortex**. Note that in this tissue from an ~2 month old animal, the zona intermedia is very sparse. There is a marked differentiation between the glomerulosa and the fasciculata, emphasizing the precision with which the signals that determine positioning of the different cell types must operate (see text).

It is important to define the terms we use. Zonation is a descriptive term, which has been applied by different observers to mean sometimes quite different things. Without a strict, generally adhered-to definition, it is most appropriate to go back to the pioneers of the terminology, and follow their usage. Since the earliest descriptors of adrenocortical zonation were morphological and topographical, and these alone were used for 70–80 years, it follows that, without a contrary formal decision, agreed perhaps by a caucus of international exponents of the field, zonation remains defined by cellular morphology and topology alone (Figures 1, 2).

The names of the three main zones are descriptive of the arrangement of their cells. Thus, the zona glomerulosa is so called because the whorls or baskets of cells that compose it somewhat recall the glomeruli of the kidney (Latin *glomus*, ball) though this is more apparent in some species (e.g., the dog) than others (rat, human). The cells of the zona fasciculata lie in parallel radial cords, and the name stems originally from the Roman symbol of office, the *fasces* or bundle of birch rods (etymologically the same origin, incidentally, as *fascist*), cf. the anatomical term *fascicle* (bundle of nerves or muscle fibers), while the cords of cells of the reticularis form a network (Latin: *rete*). Essentially, the smaller, more densely staining glomerulosa cells are characterized by their lamelliform cristae, whereas the larger and paler fasciculata cells have tubulo-vesicular cristae. The reticularis cells retain tubulo-vesicular cristae, and are again more densely staining and smaller than fasciculata cells. Lipid distribution varies with the state of stimulation, but in general is greater in the inner zones. These criteria have been reviewed on many occasions (Chester Jones, 1957; Deane, 1962; Neville and O'Hare, 1981; Vinson et al., 1992b).

Thus, zonation is not described by function, nor by the presence of this or that steroid product, or molecular marker. As it happens, and as this article will make clear, function follows morphology/topology only partially. For example, in the rat adrenal, only outer zona glomerulosa cells express CYP11B2 (see Table 1 for abbreviations) in sodium sufficient animals, and the inner glomerulosa does not. The non-CYP11B2-containing glomerulosa region was first called the white or undifferentiated zone (ZU) (Mitani et al., 1994, 1995), while others have called it the “zona intermedia” (Engeland and Levay-Young, 1999; Ulrich-Lai et al., 2006). This is confusing, because the term zona intermedia was previously applied to a morphologically distinct flattened cell type, quite different from the glomerulosa, that is limited to a very few cells at the glomerulosa-fasciculata border (Cater and Lever, 1954; Chester Jones, 1957; Deane, 1962; Nussdorfer, 1986). Indeed Cater and Lever (Cater and Lever, 1954) report it as absent from 2 month old rat adrenals, while it is very marked in older animals. It is sparsely evident in the illustration in Figure 2. It should be noted that these descriptions apply to the rat adrenal, and the presence of a discrete ZU in other species is not clear (see below).

**Table 1:** **Abbreviations used**.

ACTH	Adrenocorticotrophic hormone, corticotrophin
AgRP	Agouti-related peptide
AKR1B7	Aldo-keto reductase family 1 member B7
AKR1C3	Aldo-keto reductase family 1 member C3, type 5 17β-HSD
ANP	Atrial natriuretic peptide
AP-1	Activator protein 1 (c-fos, c-jun heterodimeric transcription factor)
APCC	Aldosterone producing cell clusters
ARNTL	Aryl hydrocarbon receptor nuclear translocator-like protein 1, = BMAL
AsP	Adrenal secretory protease
AT1R	Angiotensin II type 1 receptor
AZ1	Adrenocortical zonation factor
BMAL	basic helix-loop-helix/PER-ARNT-SIM (bHLH/PAS) transcription factor, = ARNTL, MOP3
BMP	Bone morphogenetic protein
3β-HSD	3β-hydroxysteroid dehydrogenase
11β-HSD	11β-hydroxysteroid dehydrogenase
20α-HSD	20α-hydroxysteroid dehydrogenase
18-OH-DOC	18-hydroxydeoxycorticosterone
BrdU	Bromodeoxyuridine
c-AMP	cyclic adenosine monophosphate
c-fos	See AP1
c-jun	See AP1
CLOCK	Circadian Locomotor Output Cycle Kaput gene
CRF	Corticotrophin releasing factor
Cry2	Cryptochrome circadian clock 2 gene
Cyt B5	Cytochrome B5
CYP2D16	Cytochrome P450_2D16_
CYP11A	Cytochrome P450_scc_(cholesterol side chain cleavage)
CYP11B1	Cytochrome P450_11B1_(11β-hydroxylase)
CYP11B2	Cytochrome P450_11B2_ (aldosterone synthase)
CYP17	Cytochrome P450_17_(17-hydroxylase)
CYP21	Cytochrome P450_21_(21-hydroxylase)
Dab-2	Disabled homolog-2, = DOC2, C9, p96/p67
DACH-1	Dachshund family transcription factor
DAX1	Dosage sensitive reversal adrenal hypoplasia critical region, chromosome X, = NrOb1
DHCR24	24 dehydrocholesterol reductase, seladin-1
DHEA	Dehydroepiandrosterone
Eph	Ephrin receptor
ERK	Extracellular signal regulated kinase, = MAPK
bFGF	basic Fibroblast growth factor
GATA	GATA sequence binding transcription factor
Gli1	Glioblastoma 1
Grx	Glutaredoxin
GPCR	G protein coupled receptor
IGIF	Interferon-γ inducing factor = IL 18 or IL-1γ
IGF	Insulin-like growth factor
IL	Interleukin
IP3	Inositol trisphosphate
IZA	Inner zone antigen, = PGRMC-1, Sigma 2 receptor
Ki67	Antigen K67, = MKI67
LEF1	Lymphoid enhancer-binding factor 1
LHR	Luteinizing hormone receptor
MC2R/MC5R	Melanocortin receptor 2/5 (ACTH/ melanotrophin receptors respectively)
MAPK	Mitogen activated protein kinase, = ERK
MnSOD	Manganese euperoxide dismutase
MRAP	Melanocortin-2-receptor accessory protein
α-MSH	α-melanocyte stimulating hormone
Nek2	NMA (never in mitosis gene a) related expressed kinase 2
NGFIB	nerve growth factor induced clone B;
NPY	Neuropeptide Y
Notch1,2	Notch (Drosophila) homolog 1,2
NOV	Nephroblastoma overexpressed, = CCN3
NURR-1	Nur-related factor 1
OAT	Organic anion transporter
OATP	Organic anion transporter polypeptide
ODC	Ornithine decarboxylase
Per	Period component of Per/Tim (timeless) heterodimer
Pik3c2g	Phosphatidylinositol-4-phosphate 3-kinase C2 domain-containing gamma polypeptide gene
PKC	Protein kinase C
Prkar1α	Type 1 α-regulatory subunit of cAMP dependent protein kinase
PPARγ	Peroxisome proliferator activated receptorγ
Pref1	Preadipocyte factor 1, = dlk1 (delta like homolog 1)
Prx	Peroxiredoxin
ROS	Reactive oxygen species
R26 YFP	Rosa 26 yellow fluorescent protein
Rev-Erbα)	Nuclear receptor subfamily 1, group D, member 1, NR1D1
SCN	Suprachiasmatic nucleus
SF1	Steroidogenic factor 1 (also AD4BP, NR5A1)
Shh	Sonic hedgehog (hedgehog signaling pathway component)
SLC27A2	Solute carrier family 27 (fatty acid transporter), member 2
StAR	Steroidogenic acute regulatory protein
SULT2A1	Steroid sulfotransferase
TASK1,3	TWIK-related acid-sensitive K ^+^channels, KCNK3 and 9 (members of family of two-pore domain potassium channels)
TGFβ	Transforming growth factor
TNF	Tumor necrosis factor
Trx	Thioredoxin
TrxR	Thioredoxin reductase
TSPAN12	Tetraspanin 12
Ucn3	Urocortin 3
VIP	Vasoactive intestinal peptide
Wnt	Wingless related integration site
ZU	Undifferentiated zone

Similarly, others have interpreted, and used, CYP11B2 as a “highly specific zona glomerulosa marker” (Freedman et al., 2013). It isn't, since (as noted above) it is expressed by only a small population of glomerulosa cells.

In yet another re-writing of the literature on adrenocortical zonation, some authors appear to define the zona reticularis, not on the basis of its morphology, but as the site of DHEA and androstenedione production. Defined in this way, the rat adrenal, it is claimed, does not contain a zona reticularis (de Joussineau et al., 2012; Pihlajoki et al., 2015), when it quite obviously does!—see Figure 1—moreover reticularis cells have been isolated from rat adrenals, their steroidogenic potential analyzed, and even shown to produce androstenedione, albeit in small amounts (Bell et al., 1978, 1979), see also (Pignatelli et al., 2006).

## Origin of the adrenal cortex, cell proliferation, and migration

The adrenal cortex originates from a section of the coelomic epithelium, firstly as part of the SF1-expressing adrenogonadal primordium, then separating as the adrenal anlage into approximately its ultimate location, ventrolateral to the dorsal aorta. At this site, it is invaded by cells from the neural crest, and eventually becomes encapsulated as the discrete adrenal gland (Xing et al., 2015). An interplay between the transcription factors SF1 and Dax1 determines the extent to which steroidogenic enzymes are induced, and adrenocortical cells become differentiated: Dax 1, as a co-repressor of SF1, sustains the pluripotential stem cell-like phenotype (Xing et al., 2015).

In the established cortex, the central concept that there is a stem cell population located in the capsule or immediate subcapsular region (Zwemer et al., 1938; Lombardo and Cortesini, 1988) has largely been supported by evidence from several types of experiment that track the fate of labeled cells.

Earlier studies followed labeled cells after the pulse administration of tritiated thymidine in the rat (Wright, 1971; Wright et al., 1973; Zajicek et al., 1986), latterly BrdU was used in mice (Chang et al., 2013). These gave broadly similar results, showing initial labeling in the outer part of the gland, with centripetal migration and phenotype change at a rate of 13–20 μm per day in the mouse adrenal (Chang et al., 2013), reaching the medulla after 104 days from initial labeling in the rat gland (Zajicek et al., 1986). Others have used transgenic mice bearing the 21OH/LacZ gene (Morley et al., 1996) or other constructs (Walczak and Hammer, 2015). Using 21OH/LacZ transgenic mice gives typical results in that mice bearing this transgene develop stripes of beta-gal staining cells in the adrenal extending from the capsule to the medulla, consistent with the concept of an outer region of proliferation with subsequent inward migration. It has to be said that the staining is not consistent with the distribution of 21-hydroxylase in the normal mouse adrenal, which is present only as a trace in the glomerulosa, though strongly expressed in the adrenal inner zones (Chang et al., 2013). Similar radial distribution of labeled cells has shown such lineages in mice bearing for example *shh*^*gfpcre*^*;R26-YFP* cells: *shh* is highly transcribed in the outer cortex in the mouse (though only in the ZU in the rat), so this parallels the results obtained with 21OH/LacZ. A similar cell lineage is seen in mice with the *Gli1-creT2 R26-X* label: *Gli1* is normally transcribed in the inner capsule in cells that apparently represent stem cells. Since the capsular expression of Gli1 is induced by shh signaling, this suggests what King and co-authors call a bootstrapping loop, by which the recruitment of new cortical cells from the capsule is stimulated, and both *shh* and *Gli1* cells are transformed into other cell types throughout the cortex (King et al., 2009). In fact this process is reinforced by the glomerulosa specific Dlk1 (also known as Pref-1), a member of the Notch family, which is co-expressed with shh in the rat, and, like shh, also signals to the capsule, inducing Gli-1 transcription and cortical cell recruitment (Guasti et al., 2013b; Finco et al., 2015). In mouse adrenals regenerating after dexamethasone treatment, fasciculata cells were similarly shown to be formed directly from CYP11B2-Cre-bearing zona glomerulosa cells in an SF-1 dependent process (Freedman et al., 2013), although the possibility of an intermediate shh-expressing form does not seem to be excluded.

There is often some ambiguity in such studies in that although the Standard Model is supported, alternative lineages are not necessarily excluded. Thus, fasciculata cells may be formed in the mouse adrenal from sources independent of the glomerulosa (Freedman et al., 2013). That proliferation may occur at various sites in the gland (Payet et al., 1980; Kirillov and Popova, 1985; McEwan et al., 1996, 1999) does not of itself counter the view that the stem cells are capsular or subcapsular. However, there is some additional complexity revealed by the timed appearance of BrdU labeled cells following pulse labeling in the mouse adrenal cortex that suggests there may be variation in the fates of individual cells (Chang et al., 2013), for example, some cells may be retained in the glomerulosa. Perhaps this may be expected from the evidence of the expanded glomerulosa, and CYP11B2 expression, that results from a low sodium diet or angiotensin II treatment (McEwan et al., 1996, 1999). In control mice, Ki67 staining showed proliferation predominantly in the glomerulosa and also in the outer fasciculata, where it was significantly increased by a pulse of ACTH. These fasciculata cells also expressed 21-hydroxylase, whereas the BrdU and Ki67 stained glomerulosa cells were steroidogenically inert, confirming that proliferation occurs in differentiated as well as partially differentiated cells (Chang et al., 2013).

**Figure 3:**
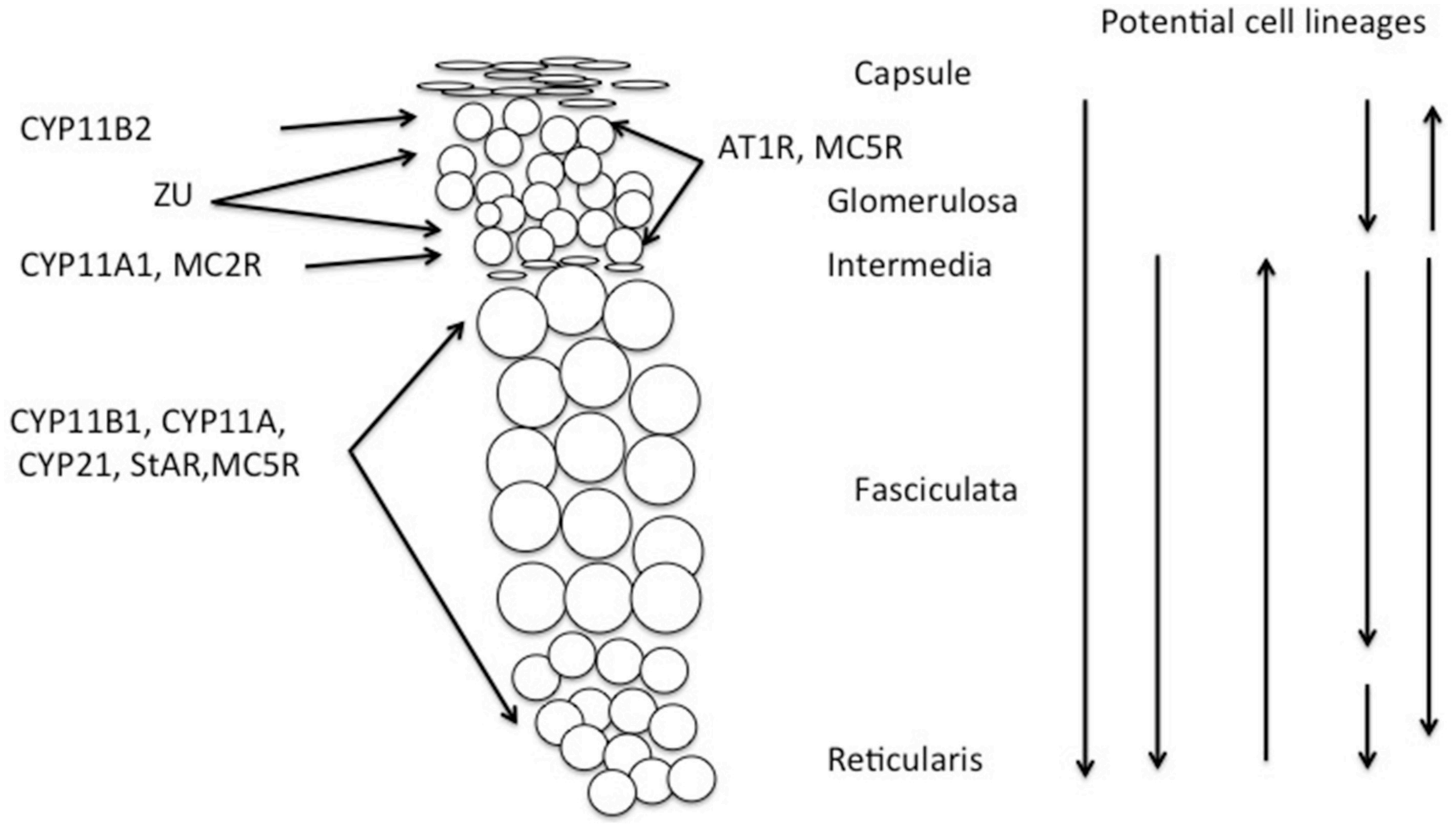
**Schematic showing distribution of melanocortin receptors and steroidogenic enzymes in the outer adrenal cortex of the rat (cf. Figure 1)**. This illustrates that cellular interaction appears to be required to explain both the synthesis of aldosterone and its regulation by ACTH, thus requiring modification of the Standard Model of adrenocortical zonation. Vertical arrows indicate potential origins of proliferation and direction of migration of adrenocortical cells. Although the stem cell population is usually held to be in the capsular/subcapsular region, evidence for all of these possible sites for subsequent proliferation has been reported, (see text and Kim and Hammer, 2007; Chang et al., 2013; Freedman et al., 2013).

Another example of such ambiguity is found in rats, where the compensatory hypertrophy following unilateral adrenalectomy may depend on proliferation in the fasciculata (Engeland et al., 2005) thus 5 days after enucleation proliferation may occur in both the glomerulosa and the inner part of the regenerating fasciculata, and after 10–28 days in the outer fasciculata (Ennen et al., 2005). Different sources of cells may also arise in other experimental conditions, for example in the Prkar1a knockout mouse, there is a strong ACTH-independent centrifugal expansion of corticosterone secreting cells, apparently from the X-zone (Sahut-Barnola et al., 2010; de Joussineau et al., 2012). All these potential sites for proliferation and migration of cells are indicated in Figure 3.

The assumption that, since adrenocortical cells originate in the outer part of the gland, and migrate centripetally, apoptosis is essentially a reticularis event, also requires re-examination, and it now appears that apoptosis occurs throughout all three zones in humans (Wolkersdorfer and Bornstein, 1998), and in the rat (Petrovic-Kosanovic et al., 2013). Like the cortex-wide proliferation that has been described, this presumably confers flexible tissue remodeling at all levels in the gland. ACTH inhibits apoptosis, thus stabilizing cortical structure *in vivo* (Carsia et al., 1996; Thomas et al., 2004), and *in vitro* according to some authors (Carsia et al., 1996) although not according to others, who describe ACTH-induced apoptosis in cultured rat cells (Mattos et al., 2011).

## Maintenance of zonal differentiation

Here zonal differentiation implies the anatomical and functional features that distinguish the cells of the different zones. As emphasized above, and will be seen in Table 2, functional differences are not always definitive, many properties may be shared. Nevertheless, it is remarkable how the differentiation between glomerulosa and fasciculata, for example, is maintained (cf. Figures 1, 2). The selection and maintenance of cellular phenotype clearly depends on location within the gland. Thus, the three adrenocortical zones always occupy the same relative position, glomerulosa outermost, reticularis innermost, adjacent to the medulla, and the fasciculata between. How can this be achieved? As discussed further below, differentiated zonal function is maintained by systemic factors, including (in experimental animals at least) angiotensin II, potassium ions and α-MSH in the glomerulosa, and ACTH in the fasciculata and reticularis. At the same time these activators are clearly not the originating stimuli for differentiation since their receptors are already zonally distributed in order for them to have a zonally specific response—they therefore reinforce the actions of existing morphogens. An example of how this can be disturbed is given by experiments in which the disruption of the tandem potassium channels TASK1 and TASK3 gives rise to extended expression of CYP11B2 in the mouse fasciculata, resulting in aldosterone secretion that is insensitive to sodium intake, though sensitive to ACTH regulation. This is only true of females, so this unprecedented chaotically unzoned hypersecretion of aldosterone is remediable by testosterone treatment (Heitzmann et al., 2008). In contrast to the action of testosterone, which tends to restore the normal zonal, i.e., exclusively glomerulosa, distribution of CYP11B2, inactivation of the Cav2.3 calcium channel in TASK1^−∕−^ mice reduces aldosterone to normal levels, but leaves CYP11B2 in the fasciculata (El Wakil, 2013).

**Table 2:** **Characteristics of zonal function in (a) rat (b) mouse, and (c) human adult adrenal glands**.

**(a) Rat**				
**Factor/Gene**	**Glomerulosa**	**Fasciculata/**	**Reticularis**	**References**
**STEROIDOGENESIS**				
CYP11A	+/−?	+++		Roskelley and Auersperg, 1990; Halder et al., 1998; Mitani et al., 1999; Guasti et al., 2013b; but see Engeland and Levay-Young (1999)
CYP11B1	−	+++	+/−	Ho and Vinson, 1993; Halder et al., 1998; Mitani et al., 1999
CYP11B2	+	−		Halder et al., 1998; Peters et al., 1998; Mitani et al., 1999
CYP21	+/−	+++		Chang et al., 2011, 2013
3β-HSD	+	+++		Dupont et al., 1991; Engeland and Levay-Young, 1999; Pignatelli et al., 1999
11β-HSD1	−	−	+	Shimojo et al., 1996
11β-HSD2	−	++	++	Smith et al., 1997; Atanassova and Koeva, 2012
StAR	−	+++		Peters et al., 1998, 2007
**RECEPTORS**				
ATR1	+++	−	−	Lehoux et al., 1997
*MC2R*	+/− Inner ZU,	+++	+/−	Gorrigan et al., 2011
*MRAP*	+/− see text	+++	+/−	
*MRAP2*	=/−	+/−	+/−	
MC5R	+++	+		Griffon et al., 1994; van der Kraan et al., 1998
**ZONAL HOMEOSTASIS**				
*Shh*	+++	−		Guasti et al., 2011, 2013b
Pref-1/dlk1	+++	−		Halder et al., 1998; Raza et al., 1998; Whitworth and Vinson, 2000
**NEUROTRANSMITTER**				
VIP	+	−		Holzwarth et al., 1987; Bernet et al., 1994
NPY	+	−		Holzwarth et al., 1987; Bernet et al., 1994; Renshaw and Hinson, 2001
Catecholamines	+	−		Holzwarth et al., 1987; Bornstein and Ehrhart-Bornstein, 1992
**CYTOKINES**				
*IGIF*	−	+++	++	Conti et al., 1997
^*^IL-6	+			Judd and MacLeod, 1992
^*^TNF	+			Judd and MacLeod, 1995
**GROWTH FACTORS**				
*Prorenin*	+	++	+	Ho and Vinson, 1998
*bFGF*	+	+		Chambaz et al., 1994; Ho and Vinson, 1995, 1997
IGF	+	+		
TGFβ	−	−		
**SIGNALING**				
ANP	+++	−		Lai et al., 2000
*AgRP*	−	+++	+++	Bicknell et al., 2000
*Prorenin*	−	+		Ho and Vinson, 1998
MAPK:ERK-1, ERK-2	++	−		McNeill et al., 1998; McNeill and Vinson, 2000; Vinson et al., 2000; Guasti et al., 2013b
SF-1	++	++		Raza et al., 1998
Dab2	+++	−		Romero et al., 2007
Nek 2b	+	+++	−	de Mendonca et al., 2014
Notch1	+++	++	+	
Notch2	+capsule	−	−	
Notch3	+++	++	+	
**DETOXIFICATION**				
**MnSOD	−	++		Raza and Vinson, 2000
**CLOCK GENES**				
*PER 1*	(?)	+++ (reticularis)		Fahrenkrug et al., 2008
*PER 2 Bmal 1*				
**OTHER**				
IZA	−	+++	++	Barker et al., 1992; Halder et al., 1998; Vendeira et al., 1999; Raza et al., 2001
*OAT1*	−	++		Beery et al., 2003
*Oatp1,2*	−	++		
*Oatp3*	++	−		
*AsP*	+++	+		Bicknell et al., 2001
*EphA2, EphA2, EphA3*	++	−		Brennan et al., 2008
Spexin	+++	++	++	Rucinski et al., 2010
		^*^Release from incubated glomerulosa cells **immunoblotting		
**(b) Mouse**				
**Factor/Gene**	**Glomerulosa**	**Fasciculata/Reticularis**	**X-Zone**	**References**
**STEROIDOGENESIS**				
CYP11B1	−	+++	− (?)	Mukai et al., 2003; King et al., 2009
CYP11B2	+++	−		
20α-HSD	−	−	++	Hershkovitz et al., 2007
3β-HSD	+++	+++	−	
CYP21	+/−	+++		Chang et al., 2013
**RECEPTORS**				
ATR1	+++	−	−	Huang et al., 2013
**SIGNALING**				
*Shh*	+++	−		King et al., 2009
*Dax1*	+++	−		Kim et al., 2009
				Mukai et al., 2002; Scheys et al., 2011
*GATA4/GATA6 GATA4 in outer capsule only*	+++			
β-catenin	+++	+/−		Tevosian et al., 2015
				Pihlajoki et al., 2015
**CLOCK GENES**				
*Bmal1, CLOCK, Cry2*	+++	++		Oster et al., 2006
*Per1, Per2, Per3, Rev-Erbα*				
*Pik3c2g*	−	−	+++	Pihlajoki et al., 2013
**DETOXIFICATION**				
AKR1B7	−?	++		Aigueperse et al., 2001; Martinez et al., 2001
Grx 1	+/−	+++	+	Godoy et al., 2011
Grx 2,5	++	+	++	
Prx 1,4,5	+	+++	+	
Prx 3	+	+	++	
Trx 1,2	+	+++	+	
TrxR2	+	−	+	
*Other*				
AZ-1	+++	++(?)	++(?)	Mukai et al., 2003; Bastida et al., 2007; Li et al., 2007
ODC	+++	++	−	
**(c) Human**				
**Factor/Gene**	**Glomerulosa**	**Fasciculata**	**Reticularis**	**References**
**STEROIDOGENESIS**				
CYP11B1	−	+++	+++	Nishimoto et al., 2010
CYP11B2	+	−	−	
^†^CYP11A		+++	+++	Rege et al., 2014
CYP17		+++	+++	Nishimoto et al., 2010
CYPB5		+	+++	Rainey et al., 2002
SULT2A1		+	+++	Rainey and Nakamura, 2008
3β-HSD	+	+++	+/−	Nishimoto et al., 2010; but see Rainey et al. (2002)
^†^*3β-HSD2*		+++	+	Rege et al., 2014
^†^*AKR1C3*		+		
**ZONAL HOMEOSTASIS**				
*DACH1*	+	+++		Zhou et al., 2015
BMP4	+++	++	+	Rege et al., 2015
**SIGNALING**				
^*^^†^*LEF1*		+++	+/−	Rege et al., 2014
^*^^†^*NOV*		+++	+/−	
^†^*SLC27A2*		+	+++	
^†^*TSPAN12*		+	+++	
NGFIB	+++	++		Bassett et al., 2004b
Nurr-1	+++	+		
PPARγ	++	+		Uruno et al., 2011
Ucn3	+	+		Fukuda et al., 2005
CRF1	+/−	++	++	
CRF2	+/−	++	++	
LHR		+/−	+	Pabon et al., 1996
**DETOXIFICATION**				
Seladin	−	+++	+++	Battista et al., 2009
^†^*AKR1C3*		+	+++	Rege et al., 2014

*This table lists some of the genes and gene products that have been used to define the different functions of the zones of the adrenal cortex, mostly using either immunocytochemistry (shown in normal text) or in situ hybridization (in italics). It is by no means exhaustive, many more components have been listed, notably when cDNA arrays have been used, (e.g., de Mendonca et al., 2014; Nishimoto et al., 2014; Tyczewska et al., 2014). Plus signs (+) indicate presence, minus signs (−) absence. The number of + signs gives a rough indication of the abundance of signal under control conditions, i.e., with no special treatment. Naturally all of these values may change, sometimes enormously, if neutral physiological regimes are perturbed*.

* *Also reported in glomerulosa, but data not provided*.

† *Transcriptome profiling*.

So, if systemic factors cannot be responsible for localization of cell type and the organization of the zones, these must instead depend on factors within the gland. It may be speculated what such factors might be. We have previously considered these to be of three types, morphogens or paracrine agents produced within the gland, growth factors that essentially amplify the morphogenic signal, and transcription factors (Vinson and Ho, 1998; Vinson, 2003). In essence this is illustrated by the action of shh in upregulating Gli1, or the Wnt/catenin systems, described more fully below.

The concept that morphogens, secreted by a fixed source, diffuse along a concentration gradient to inform more or less remote cells of their position, and trigger appropriate responses, is sometimes difficult to envisage, because of the randomness of diffusion, and its susceptibility to local variation due for example to tissue inhomogeneity (Lander, 2013), and, in the case of the adrenal, also because of the relatively massive centripetal blood flow (Vinson et al., 1985b; Vinson and Hinson, 1992). The actions of morphogen gradients have been most widely studied in the context of limb development and regeneration in mice and other vertebrates. In mice, for example, bone morphogenetic proteins, BMPs, which are closely related to the TGF family, stimulate local growth and regeneration (Urist, 1965; Reddi, 1998; Bandyopadhyay et al., 2006; Tsuji et al., 2006; Yu et al., 2012) and have been primarily recognized for their roles in stimulating chondrogenesis and osteogenesis. However, they are also important in other tissues including the prostate (Thomas et al., 1998) breast and prostate cancer (Buijs et al., 2010), and in the pituitary and other components of the reproductive system (Shimasaki et al., 2004). Here wnt and shh signaling is crucial, as in the early development of the adrenal (below). In the limb, induction of shh is brought about by retinoic acid, which generates positional information at various stages in development (Tickle, 2006). This is achieved with a robustness that is remarkable, given the inherent variation in retinoic acid availability, depending as it does on dietary sources. But retinoic acid gradients may not depend solely on release and diffusion, but additionally on sites of degradation, as in the zebrafish brain (White et al., 2007; Schilling et al., 2012). It is possible that this applies to other morphogens, which could be critical when not only morphogen concentration but the duration of target cell exposure is critical, as in the mouse limb response to shh (Tickle, 2006). Crucially, it is thought that sharp boundaries between different cell types, such as found in the adrenal cortex, can still be achieved in the presence of a fluctuating and shallow morphogen gradient, by means of various mechanisms that depend in part on cell plasticity and in part on intercellular signaling. In other systems, these may include for example the retinoic acid-sensitive autoactivating genes Krox 20 and Hoxb1 that are mutually inhibitory in the zebrafish hindbrain (Zhang et al., 2012). Other mechanisms of noise-induced switching have been described (Schilling et al., 2012). Nevertheless, interfering with gap junctions affects positional clarity (Tickle, 2006), so direct cell-cell signaling is also involved (Lander, 2013).

With regard to the adrenal, it is clear that the same morphogens may play a role as in vertebrate limb regeneration, notably Wnt, Shh and BMPs. BMPs are expressed in all zones of the human adrenal cortex, but particularly the glomerulosa (see Table 2). Additionally, in H295R cells, BMP4 suppresses CYP17 RNA and protein, and DHEA secretion (Rege et al., 2015). In contrast, overexpression of the transcription factor DACH1 activates TGFβ and canonical Wnt signaling pathways, thereby suppressing CYP11B2 and aldosterone secretion (Zhou et al., 2015). Considerable advances in recent years have highlighted the particular importance of the Wnt/catenin system (MacDonald et al., 2009). Secreted Wnt glycoproteins bind to GPCRs of the *frizzled* family, bringing about increased cell content of the coactivator β-catenin, now known to be essential for the differentiation of SF1 positive adrenocortical cells from the adrenal capsule, and for their proliferation (Kim et al., 2008, 2009). The Wnt/catenin signaling system is involved in adrenocortical development in other ways also, by interaction with the nuclear receptor Dax1 and inhibin, both of which modulate adrenal/gonadal selection. Dax1is induced by SF1, but inhibits SF1 stimulated gene transcription (Ito et al., 1997; Yu et al., 1998). DAX1 is prominent in the subcapsular region in the mouse adrenal (but completely absent from the X-zone) (Mukai et al., 2002), together with β-catenin (Pihlajoki et al., 2015). Inhibin-α, which suppresses gonadal phenotype, is induced by SF1 and β-catenin (Gummow et al., 2003). The overall outcome of these effects in combination, is both to establish the adrenocortical fate of the subcapsular cells, while maintaining their further developmental plasticity (Wood and Hammer, 2010; Simon and Hammer, 2012). This, in effect, defines the glomerulosa (Berthon et al., 2012). SF1 is absolutely required for the development of the gland, and for the cell redifferentiation associated with centripetal migration (Freedman et al., 2013).

The search for stimulators of migration has however so far produced meager results. It is possible that the extracellular matrix may be involved: in bovine adrenal cells, laminin secretion is regulated by ACTH, and, in Boyden chamber experiments, laminin acted as a chemoattractant in promoting adrenal cell migration. However, the distribution of laminin is uniform throughout the cortex, and therefore seems unlikely to promote adrenocortical cell cellular migration (Pellerin et al., 1997). Other extracellular matrix proteins, including fibronectin and collagens I and IV, have been shown to affect steroidogenic enzyme expression in rat adrenal cells in culture, and in particular to modulate basal and ACTH stimulated steroid secretion (Otis et al., 2007). Additionally, rat glomerulosa cells secrete fibronectin, which promotes proliferation, while angiotensin II interferes with this process at the level of integrin binding, thus inhibiting proliferation while enhancing steroidogenesis (Otis et al., 2008).

**Figure 4:**
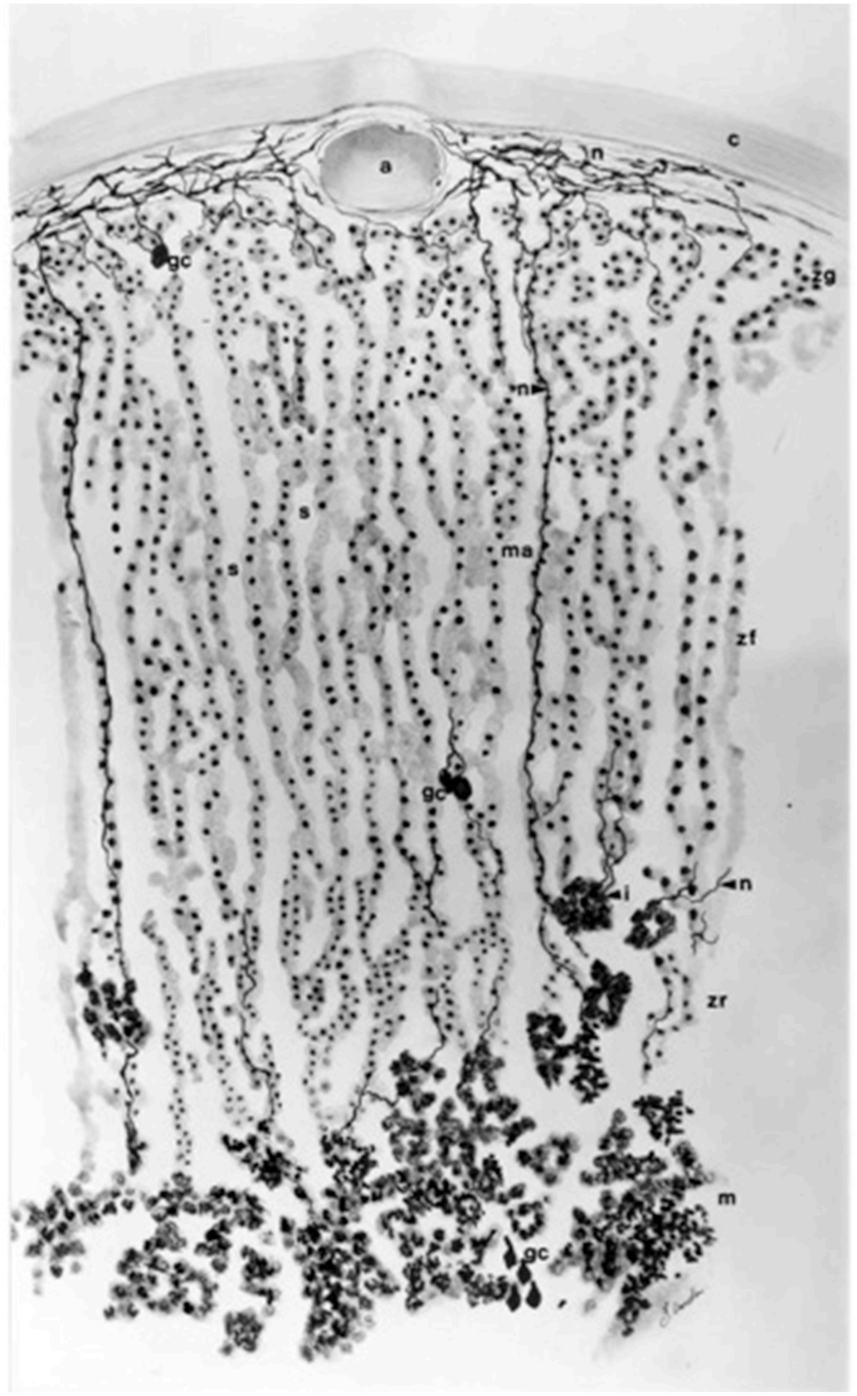
**General arrangement of the vasculature and innervation of the rat adrenal cortex**. Most nerves are located in the capsular (c) and subcapsular region, with arborization around the vasculature, including arterioles (a) and around the cells of the glomerulosa (zg). Nerve fibers are rare in the fasciculata (zf), but some traverse the width of the zone, often associated with medullary arteries (ma, 6–8 per gland), which specifically supply the medulla. Most of the blood reaching the medulla passes centripetally through the sinuses, which, in contrast to the thicker walled medullary arteries, are bordered by a single layer of very attenuated endothelial cells (cf. Figure 2). Near the medulla (m) a few short nerve fibers reach the reticularis (zr) from islets of chromaffin cells (i) in the inner cortex and from the medulla itself. There is now considerable evidence that products from both neural tissue and vasculature can affect corticosteroid secretion. They may also provide positional information for migrating and transforming cortical cells (see text). Drawing by Bridget Landon. Reproduced with permission from Vinson et al. (1994).

Equally, how the inward migration of cells is achieved while the position of the zones relative to each other remains constant also continues to be a matter of some mystery. It is possible to speculate that various mechanisms might play a role. It is entirely possible that structures within the gland external to the adrenocortical cells themselves provide positional information. Such structures could include the neural network or elements of the vasculature, which though not necessarily visible in conventional microscopy are nevertheless important components both of which affect adrenocortical function (Figure 4; Vinson and Hinson, 1992; Vinson et al., 1994) and may be independent of ACTH (Bocian-Sobkowska et al., 1997). Alternatively, the relative positions of the capsule, external to the cortex, and of the inner medulla might suggest that a morphogenetic landscape could be generated by signals from these two poles. Certainly medullary products can affect inner cortical function (Ehrhart-Bornstein et al., 2000; Haidan et al., 2000). Or, another possibility is that positional information is provided by the adrenocortical cells themselves. Cortical cells, arranged as they are in cords traversing the cortex, themselves provide a polarity, indeed each cell may have an apical and a basal pole. Clearly this is true at least of some of them: cells of the outermost glomerulosa layer (which express both CYP11B2, AT1R, and MC5R) about the capsule at one pole, and other, perhaps less differentiated cells at the base. The innermost glomerulosa cells in the ZU abut either true zona intermedia or outer fasciculata cells, while the innermost reticularis cells lie directly adjacent to the medulla in the rat: in other species, such as the postpubertal male mouse there may be a band of connective tissue around the medulla. Such cell-cell contact is known to be important in positional information in other tissues (Tickle, 2006; Lander, 2013). The Eph/ephrin forward-reverse signaling system provides a possible mechanism for such cellular orientation and migration within the zones, and elements of the system have been demonstrated in the rat adrenal cortex. In particular, EphA2 is strongly transcribed in the rat glomerulosa, and responds to the physiological stimuli of a low sodium diet, captopril, or betamethasone treatment in a manner that suggests association with cell phenotype (Brennan et al., 2008).

## Functional zonation of the adrenal cortex

A summary of some of the components expressed in the zones of the rat mouse and human cortex is given in Table 2. The Standard Model of adrenocortical zonation, as summarized in the Introduction, interprets functional zonation in terms of steroid hormone output alone. It is quite evident that in fact many other adrenocortical cell products are zone specific, and functional zonation is not just about steroids. Many of these other zonally organized functions, such as receptors and signaling pathways that transduce trophic hormone action, can be considered to complement the steroidogenic function. Others, such as clock genes, or enzymes metabolizing xenobiotics, may not be quite so easily accommodated in this way.

Although the same corticosteroids, cortisol, corticosterone, and aldosterone (though not usually all three) appear throughout the non-mammalian vertebrates, functional zonation of the adrenal cortex is a universal feature in mammals alone. While the appearance of the zones in different mammalian species may vary, aspects of their function do not. Thus, it is clear that the CYP11B2 catalyzed production of aldosterone is invariably limited to a population of cells in the zona glomerulosa, and CYP11B1 generates cortisol (or corticosterone in rats, mice, and some other species) predominantly in the fasciculata, and to a lesser extent, in the reticularis. Though this is consistent with the Standard Model, it is nevertheless now clear that some qualification is required.

The qualification arises because the extent of *de novo* steroidogenesis that can be unequivocally associated with each zone is limited. The human adrenal, for example, is complex. Here the well-defined CYP11B2-expressing glomerulosa seen in young individuals gives way in advancing years to a sparse glomerulosa generally confined to scattered islets (Neville and O'Hare, 1981; Aiba and Fujibayashi, 2011), interspersed by cells that may be analogous to those of the rat ZU [and are called the “progenitor zone” by Aiba and Fujibayashi (2011)], but which express 3β-HSD. Later, CYP11B2 expressing cell clusters (APCC) develop at the glomerulosa/fasciculata border, and extend into the fasciculata (Aiba and Fujibayashi, 2011; Nishimoto et al., 2015): these appear from the age of about 30 years. These additional sites of aldosterone production, arising as a consequence of somatic mutation of ion channels, are common in normal subjects, but the authors hypothesize that they predispose to primary aldosteronism (Nishimoto et al., 2015).

In the rat zona glomerulosa, starkly, of the steroidogenic enzymes only CYP11B2 is at all strongly expressed, and that only in the outermost glomerulosa: other required components for complete steroidogenesis, including CYP11A and CYP21 are barely detectable (Table 2), as is StAR according to some authors (Peters et al., 1998, 2007), but not others (Lehoux et al., 1998; Tyczewska et al., 2014). 3β-HSD appears to be present in all zones in the rat, as it is in the mouse (Table 2) though it is not expressed in the glomerulosa of the bovine adrenal (Ishimura and Fujita, 1997) while in humans it is relatively sparse in the glomerulosa according to some authors (Nishimoto et al., 2010), but not others (Rainey et al., 2002). Guasti et al. (2013a) located CYP11A in the inner undifferentiated zone (ZU) of the rat adrenal zona glomerulosa, quite clearly—indeed by definition—not a site of CYP11B2 expression, which, as noted, is normally confined to a very few cells in the outer glomerulosa. Notably, this site of CYP11A expression coincides with an intense signal for MC2R mRNA, otherwise apparently lacking in the rat glomerulosa (Gorrigan et al., 2011). The intensity of MC2R mRNA staining in this region is not inconsistent with the observations of Gallo-Payet and Escher (1985) who found greater ACTH binding in the glomerulosa than other zones, but who did not distinguish between CYP11B2 expressing and non-expressing glomerulosa cells. Also contrary to expectation, StAR and CYP11A are not always co-expressed. Thus not only does the inner ZU site of CYP11A not coincide with CYP11B2, with the caveats above in mind, it does not here coincide with StAR either: Peters and colleagues have consistently demonstrated StAR expression throughout the fasciculata, but, in untreated animals, hardly at all in any of the morphologically distinct smaller glomerulosa cells in the rat, including the ZU (Peters et al., 1998, 2007).

SF1 though is very definitely present throughout the adrenal cortex (Morohashi et al., 1994; Raza et al., 1998). Although SF1 is absolutely required for the development of the gland and its steroidogenic capacity (Lala et al., 1992; Parker and Schimmer, 1993; Parker et al., 1995; Freedman et al., 2013), its role must be very different in the cells of the different zones. Thus, although exposure to high SF1 inhibits CYP11B2 expression (Ye et al., 2009), the presence of SF1 in the glomerulosa is required not only for functional CYP11B2, but also for the glomerulosa-fasciculata cell type transformation that is a feature of adrenal cell centripetal migration and redifferentiation (Freedman et al., 2013).

In contrast to the glomerulosa, all fasciculata cells strongly express all of the enzymes required for glucocorticoid production enzymes, CYP11A1, CYP21, CYP11B1, 3β-HSD, and StAR, together with, in human (and other species') glands, CYP17 as well. These components are also present in the reticularis though (with the possible exception of CYP17) in lower abundance.

This then raises the question: how is aldosterone produced in the rat and human gland, if the entire required suite of enzymes and StAR are not co-expressed? Twenty years ago, we suggested that (at least) two cell types are required for aldosterone synthesis and secretion in the rat adrenal (Vinson et al., 1995; Vinson, 2004). The evidence for this is primarily that in the rat the efficient production of aldosterone *in vitro* requires an intact gland, and as it is disrupted, from whole tissue, first to separated zones, then to enzyme dispersed cells, aldosterone yields *in vitro* are progressively diminished, whereas corticosterone output is unchanged, and 18-OH-DOC, a major product of the fasciculata in the rat (Tait et al., 1970; Nonaka and Okamoto, 1991; Okamoto and Nonaka, 1992), is increased (Vinson et al., 1985a). Together with other evidence this suggested 18-OH-DOC might be an aldosterone precursor in this species (Vinson et al., 1992a, 1995; Vinson, 2004). Objections may be raised on the basis that the hemiketal structure of 18-OH-DOC is stable, and not easily converted, that the blood flow is centripetal, hence precluding steroid movement from inner zone to the glomerulosa, and that conversion of exogenous precursors is invariably inefficient. However, tracer 18-OH-DOC is certainly converted to aldosterone by rat glomerulosa tissue, and is sensitive to sodium status (Fattah et al., 1977). How it could reach the glomerulosa *in vivo* remains open to speculation, but if we accept that shh and wnt diffuse centrifugally (see above), there seems no reason why other substances should not. Or there may be cell-cell transport: it is not the proteolytic enzmes used in cell dispersal methods that diminishes aldosterone output, but the physical disruption of the capsular tissue (Raven et al., 1983). Finally, in the rat 18-OH-DOC circulates at concentrations sufficient to be utilized as substrate (Vinson, 2004).

**Figure 5:**
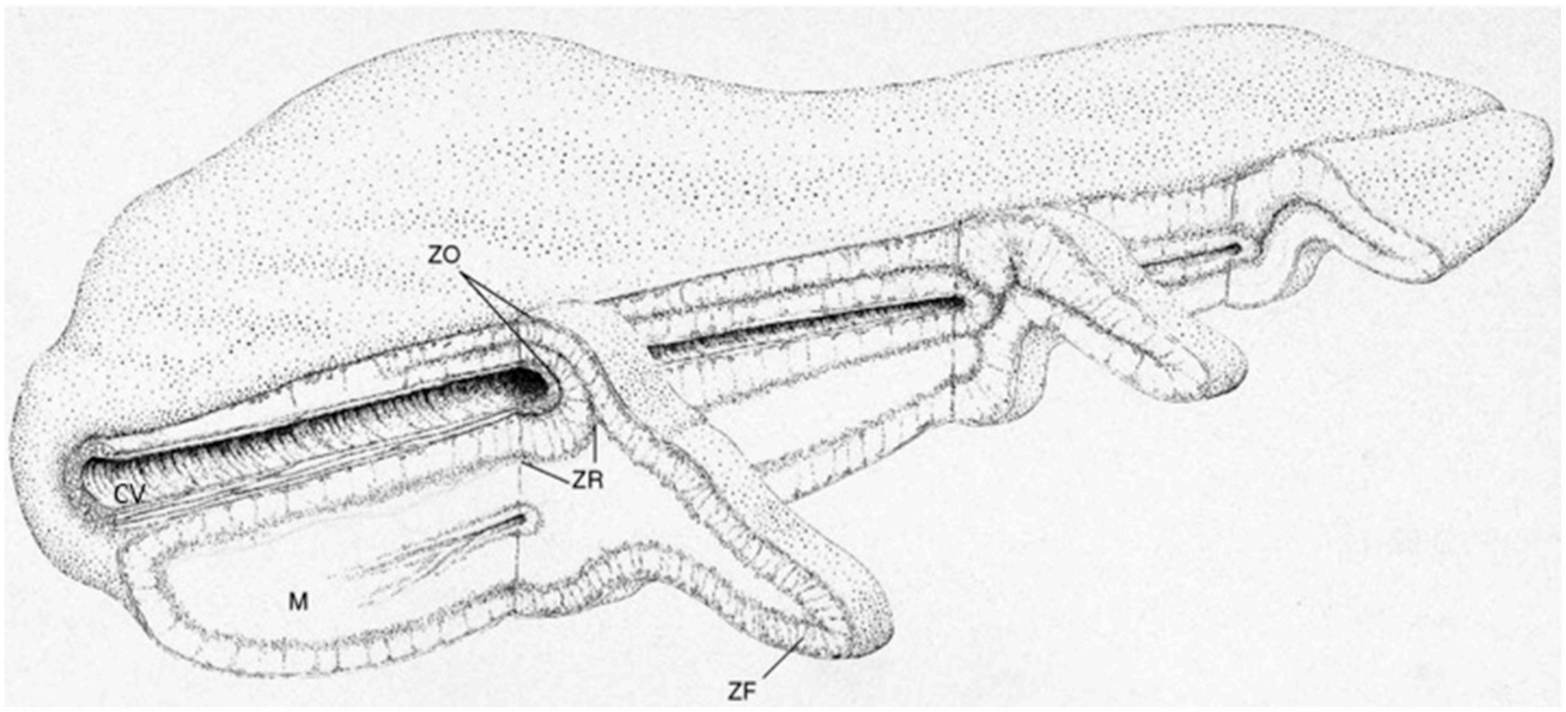
**General structure of the human adrenal gland showing the cortical cuff**. The inward folding adjacent to the central vein results in a doubling of the cortical thickness and the juxtaposition in this region of the inner zona glomerulosa and the central vein. C, cortex; M, medulla; ZG, zona glomerulosa; ZF, zona fasciculata; ZR, zona reticularis; CV, central vein. Drawing by B. Landon. Figure reproduced with permission from Vinson and Hinson (1992).

It is not suggested that the same mechanism would necessarily operate in any species other than the rat, which seems to be unique in that 18-OH-DOC is a major product of the fasciculata. Nevertheless, in this connection it is intriguing that in the human gland there exist more obvious mechanisms for the transfer of steroid substrate or other products from the inner zones to the glomerulosa. First, there are arteriovenous loops which derive from the cortical arterioles in the capsule region, pass through the glomerulosa and fasciculata, but then loop back to the outer cortex to a site near their origin. Secondly, as the adrenal vein leaves the gland, the cortex is inverted and wraps around the vein as the cortical cuff, which retains its zonation, (though inverted). Here the blood supply is from the arteriae comitantes in the wall of the vein. Drainage from this region is through vessels from the medulla through the cortex (i.e., from reticularis to the fasciculata and glomerulosa), thence to the central vein (Figure 5) (Dobbie and Symington, 1966; Neville and O'Hare, 1981). Finally, in older subjects, CYP11B2 expressing aldosterone producing cell clusters project into the fasciculata (Nishimoto et al., 2015), providing the possibility of much more intimate contact between the different cell types.

A more specific function for the reticularis has sometimes seemed to be less clear cut, especially in those species in which 17-hydroxylase (CYP17) is sparse, but in the human, and some other species, it is the major source of C_19_ steroids, especially dehydroepiandrosterone, both as the free steroid and as the sulfate. This general concept has been strengthened by methods developed to separate individual cell types, and assess their steroid output *in vitro*, and most recently by microdissection and transcriptome profiling (see Table 2). Notably, these methods have shown that CYP11A1 (side chain cleavage) and CYP17 (17-hydroxylase) gene transcripts are similar in the human fasciculata and reticularis, but Cyt B5A, sulfotransferase SULT2A1, and 17β-HSD (aldo-ketose reductase AKR1C3) are significantly more prominent in the reticularis (Rainey et al., 2002; Rege et al., 2014). The combined activity of these components accounts for the characteristic secretion of large amounts of DHEAS by the reticularis: Cyt B5 has been recognized as an important regulator of CYP17, and its presence is required for the efficient side chain cleavage activity of CYP17, though not for its 17α-hydroxylase function (Hall, 1991; Lee-Robichaud et al., 1995; Soucy and Luu-The, 2002). Cyt B5 acts through allosteric action rather than (as originally thought) provision of the second electron required for side chain cleavage (Auchus et al., 1998; Akhtar et al., 2005). It is the heightened secretion of DHEA and DHEAS by the reticularis that incurs the onset of adrenarche in humans (Belgorosky et al., 2008).

How then is androstenedione, the second most abundant androgen secreted by the human adrenal, actually synthesized? As we now see, in the human gland, the fasciculata cell expresses 3β-HSD, but the reticularis only a trace. On the other hand, the fasciculata expresses relatively little Cyt B5, required for C_21_ steroid side chain cleavage, whereas the reticularis expresses a lot. Perhaps interaction between the cell types, as postulated for aldosterone biosynthesis above, provides an answer, with transfer of DHEA from the reticularis to the fasciculata for the final stage of 3β-HSD catalyzed dehydrogenation/isomerization to give the Δ^4^-3-ketone configuration of androstenedione.

Zonation in the adrenal cortex of the mouse is more complex than the broad divisions into glomerulosa, fasciculata, and reticularis, usually taken as the mammalian norm, because of the additional presence of an inner zone, the X-zone, that is only apparent in the prepubertal animals of both sexes, and in the nulliparous adult female. It does not appear to be responsive to ACTH, and it has been suggested that it is supported by LH (Chester Jones, 1955). It involutes at puberty in the male mouse, and at first pregnancy in the female leaving a band of connective tissue between the reticularis and the medulla. Its involution is exquisitely sensitive to testosterone, and although from more recent studies it appears that involution precedes the increase in blood testosterone associated with puberty in the male (Hershkovitz et al., 2007), involution does not occur in castrates, and castration of adult males results in the development of a secondary X-zone lying outside the connective tissue residue of the original (Chester Jones, 1955, 1957). The function of the X-zone has remained largely obscure, though it is known to express 20α-hydroxysteroid dehydrogenase, but not 3β-hydroxysteroid dehydrogenase, which occurs widely throughout the rest of the cortex (Hershkovitz et al., 2007). To some extent the lack of data regarding this zone is compounded by the wide variation in its appearance, indeed in some inbred laboratory animals, notably the DDD strain, the extent of X-zone vacuolation suggests the presence of little functional tissue at all (Suto, 2012).

Other components that have a clear zonal distribution are enzymes that are thought to protect the gland against toxic products associated with a high degree of cytochrome P450 activity. Thus the aldo-keto reductase-like enzyme, AKR1B7 disposes of isocaproic aldehyde, the byproduct of CYP11A1 mediated cholesterol side chain cleavage, and manganese superoxide dismutase may protect against free radicals arising from leaky mitochondrial cytochrome P450 activity, Both of these enzymes are expressed in inner adrenocortical zones (in mouse and rat respectively) (Sahut-Barnola et al., 2000; Martinez et al., 2001; Raza et al., 2005), but not in the glomerulosa. In this context, seladin-1 (DHCR24), highly expressed in human and rat fasciculata cells, may be involved in oxidative stress management, and use of a specific seladin-1 inhibitor enhances ROS formation in response to ACTH (Battista et al., 2009). It may also be involved in specific regulation of DHEA secretion (see below).

The multifunctional protein that we originally called the Inner Zone Antigen (IZA) because it is present in the rat adrenocortical fasciculata and reticularis but not glomerulosa (Laird et al., 1988; Barker et al., 1992) was later identified with the so-called progesterone receptor membrane component 1 (PGRMC1) (Min et al., 2004; Cahill, 2007), and is now also designated as the sigma 2 receptor (Xu et al., 2011; Zeng et al., 2016). In the rat adrenal this protein specifically supports 21-hydroxylation (Laird et al., 1988; Min et al., 2005). The inner zone distribution of all three of these components, AKR1B7, MnSOD, IZA/PGRMC1/Sigma2, and their absence from the glomerulosa is entirely consistent with the view that the glomerulosa is not a significant site of *de novo* steroidogenesis.

Another metabolic function of the adrenal has been described that has a distinct zonal distribution, this is the capacity for xenobiotic metabolism conferred by CYP2D16, a member of the CYP2D cytochrome P450 subfamily, in the reticularis of the guinea pig (Martin and Black, 1983; Black et al., 1989; Jiang et al., 1996; Yuan et al., 1998, 2001). CYP2D16 increases with age, and is more abundant in males than in females, there are also strain differences (Huang et al., 1996). In contrast to CYP17, which is enhanced, CYP2D16 is diminished by ACTH treatment (Yuan et al., 1998), as is 21-hydroxylase in the reticularis, but not in the outer zones (Colby et al., 1992). It is also bimodally distributed in that it is abundant in the majority of individual animals, but low in others (Colby et al., 2000). CYP2D isoforms themselves have 21-hydroxylase activity however, and are responsible for this function in the rat brain (Kishimoto et al., 2004). Type 5 17β-HSD, another member of the aldo-keto reductase family, also designated AKR1C3, is prominently transcribed in the human reticularis (Rege et al., 2014). Its function, presumably, is the reduction of 17-ketosteroids, such as androstenedione to testosterone (Nakamura et al., 2009). Conceivably it may also be protective, perhaps, like CYP2D16, metabolizing xenobiotics or (like AKR1B7) metabolizing isocaproic aldehyde. Interestingly, in other cell types, AKR1C3 also suppresses cell differentiation (Desmond et al., 2003).

Also not directly part of the corticosteroid synthesizing apparatus are the clock genes, which are zonally distributed, though quite differently, in rat and mouse adrenals. Thus *Per 1, Per 2*, and *Bmal 1* are most strongly expressed in the reticularis in rats, but, together with other clock genes (see Table 2) in glomerulosa and fasciculata in mice. Their expression depends on clock time, and is instrumental in maintaining the circadian rhythm inherent in corticosteroid secretion, in conjunction with hypothalamic/pituitary input (and in turn its regulation by the SCN clock system) and SCN regulated autonomic innervation (Ishida et al., 2005; Chung S. et al., 2011; Leliavski et al., 2015).

The secretion of endogenous ouabain is also zonal, in that bovine glomerulosa cells secrete several times as much as fasciculata cells, both under control conditions, and also when stimulated by angiotensin II or ACTH (Laredo et al., 1995). The presence of signaling molecules NOV, SCL27A2, and TSPAN12, not previously associated with adrenal function, and their differential transcription in the zones of the human adrenal cortex (Rege et al., 2014; see Table 2) also add to the view that our understanding of zonation is far from complete.

## Regulation of adrenocortical zonation

### Trophic regulation of zonal function

In part, the maintenance of the differentiated cortex follows the activation of specific signaling pathways. Glomerulosa steroidogenic function, that is the expression of CYP11B2 and the final stage of aldosterone synthesis, for example, is induced by (among other factors) angiotensin II stimulation, via angiotensin type 1 receptors (ATR1a and b) resulting in the activation of calcium mediated signaling pathways, whereas the fasciculata, CYP11B1 expression and cortisol/corticosterone production are ACTH dependent, modulated by the melanocortin type 2 receptor (MC2R) acting through cAMP formation.

### Regulation of the glomerulosa

Angiotensin II, the potassium ion and ACTH are the most widely studied regulators of aldosterone secretion and glomerulosa function. They have both acute effects and longer term trophic actions.

There is ample evidence that angiotensin II supports glomerulosa structure and aldosterone secretion in a manner which parallels that of dietary sodium restriction: this topic has been reviewed on many occasions, and need not be repeated here (Mulrow, 1999; Hattangady et al., 2012; Bollag, 2014). The potassium ion also has direct stimulatory actions, both on acute secretion of aldosterone (Haning et al., 1970; Aguilera and Catt, 1978), and, longer term, in induction of CYP11B2 (Tremblay et al., 1992; Yagci et al., 1996). Its action is mediated through voltage gated calcium channels (Haning et al., 1970; Aguilera and Catt, 1986; Kenyon et al., 1991) but also may require the presence of angiotensin II, perhaps generated by the intra-adrenal RAS, as well (Yamaguchi et al., 1990; Tremblay et al., 1992; Gupta et al., 1995; Vinson et al., 1998), certainly the actions of angiotensin II and potassium ions are linked (Shepherd et al., 1991).

ACTH, has long been recognized as an acute stimulator of aldosterone secretion (Haning et al., 1970; Aguilera and Catt, 1978; Braley et al., 1992; Williams, 2005; Hattangady et al., 2012; Bollag, 2014). In the rat, this presents an enigma, how is this achieved?—given what we now know about the distribution of MC2R and CYP11A expressing cell types, which in the glomerulosa are seemingly confined to the inner ZU and do not coincide with CYP11B2 (Gorrigan et al., 2011; Guasti et al., 2013a). But glomerulosa preparations, such as used by many authors (Haning et al., 1970; Aguilera and Catt, 1978; Braley et al., 1992; Williams, 2005) clearly will contain both CYP11B2 and CYP11A/MC2R-expressing cell types, as well as, usually, some fasciculata cells as well. This raises the possibility of cell-cell interaction, developed further below.

Chronically, on the other hand, ACTH does not support the glomerulosa cell phenotype, but instead downregulates CYP11B2, induces CYP11B1, and in these and other ways imposes the fasciculata phenotype instead (Aguilera et al., 1981; Ho and Vinson, 1993; Ni et al., 1995; Mitani et al., 1996). Though aldosterone secretion is also sensitive to ACTH stimulation (Hattangady et al., 2012), MC2R inactivated mice have normal glomerulosa, but greatly involuted fasciculata (Chida et al., 2007), whereas POMC knock-out may disrupt the adrenal entirely (see below). This may be attributed to the additional loss of α-MSH which in rats acts specifically on the glomerulosa in which MC5R abound (van der Kraan et al., 1998; Liakos et al., 2000): α-MSH acting on MC5R is 10 times more potent than ACTH (1-39), and 100 times more than γ-MSH in stimulating cAMP (Griffon et al., 1994). There is however a problem in that α-MSH stimulation of aldosterone production by the rat adrenal is not convincingly linked to cAMP, which is only stimulated at high α-MSH concentrations, whereas IP3 and PKC pathways are activated at lower levels (Kapas et al., 1992, 1994); others have suggested that a further MSH receptor remains to be discovered (Bicknell et al., 2000). In hypophysectomised rats, α-MSH, not ACTH is the POMC product that restores aldosterone secretion and its response to sodium depletion (Shenker et al., 1985), cf. (Vinson et al., 1980; Costa et al., 2011).

Downstream of the increased intracellular calcium evoked by potassium or angiotensin II, the orphan nuclear receptors Nurr1 and NFGFIB are both implicated in CYP11B2 expression, which also responds to COUP, SF-1 and CREB through promoter sites (Bassett et al., 2004b,c). NFGFIB, somewhat more widespread through the (human) cortex than Nurr1 (see Table 2), also mediates the expression of 3β-HSD2, which converts the Δ^5^,3β-hydroxy configuration of 17α-hydroxypregnenolone or DHEA to the corresponding Δ^4^,3-ketones (Bassett et al., 2004a,d). Angiotensin II also inhibits CYP17 expression, via PKC and src-kinase mediated pathways.

Many other factors have been found to modulate aldosterone secretion, at least under experimental conditions, these include particularly the neurotransmitters (Torda et al., 1988; Janossy et al., 1998; Vinson, 2003; Whitworth et al., 2003) cytokines, which are also produced within the gland (Judd and MacLeod, 1992; Ehrhart-Bornstein et al., 1998; Judd, 1998; Call et al., 2000), vascular products, including endothelins, nitric oxide, and adrenomedullin (Hinson and Kapas, 1998; Delarue et al., 2004), and adipokines (Willenberg et al., 2008).

### Actions of ACTH on adrenal proliferation and zonation

The earliest observations on the effects of hypophysectomy and ACTH treatment on adrenal form and function, showing the absolute dependence of the inner adrenocortical zones, but not the glomerulosa, on an intact functioning pituitary, was borne out by later experiments using specific gene knockout methods. First, the elimination of POMC products, including ACTH, was shown to prevent the complete development of the adrenal gland. In one study, the adrenals of POMC^−∕−^ mice were not macroscopically discernable, and both corticosterone and aldosterone were undetectable (Yaswen et al., 1999). However, in another report, although both fasciculata and reticularis/X-zone were greatly reduced in the mutant animals, the glomerulosa was relatively unaffected, and this was reflected in the milder effect on circulating aldosterone, while corticosterone was virtually eliminated (Coll et al., 2004). The administration of ACTH restored both circulating corticosterone (but not aldosterone) and adrenocortical structure to normal (Coll et al., 2004). In a later study, MC2R^−∕−^ animals were also shown to have normal glomerulosa structure, but greatly reduced inner adrenocortical zones, combined with undetectable plasma corticosterone while aldosterone was reduced by two thirds (Chida et al., 2007). These effects, of POMC [at least in the (Coll et al., 2004) study] and MC2R knockout, appear accurately to reproduce the actions of hypophysectomy (Chester Jones, 1957; Deane, 1962; Vinson, 2003). It is curious that the rat adrenal also expresses AgRP, an α-MSH antagonist (Yang et al., 1999) but only in the fasciculata and reticularis, and not the glomerulosa (Bicknell et al., 2000): in the mouse, the agouti locus is associated with X-zone, but not reticularis, morphology (Tanaka et al., 1994, 2006).

Other studies suggest that even within the zona fasciculata different areas may respond differently to chronic stress, which induced hyperplasia in the outer fasciculata, and hypertrophy in the inner fasciculata, while glomerulosa cell size was reduced (Ulrich-Lai et al., 2006).

Peptides derived from pro-γ-MSH, a 16kD fragment from the N-terminus of the POMC molecule secreted by the pituitary, that do not contain the γ-MSH sequence are thought to have a role in stimulating adrenocortical proliferation (Estivariz et al., 1982, 1988) and POMC 1-28 in particular has been shown to have this activity. An adrenal serine protease, designated AsP, which is thought to generate mitogenic peptides from pro-γ-MSH at the adrenocortical cell surface, has been characterized (Bicknell et al., 2001; Bicknell, 2003). Support for this concept has been provided by some subsequent authors, who found that POMC 1-28 stimulated adrenocortical proliferation in dexamethasone or hypophysectomised rats, though primarily in the glomerulosa (Torres et al., 2010; Mendonca and Lotfi, 2011), but others found it had no effect on adrenal growth in POMC^−∕−^ mice, although there was a good response to ACTH (Coll et al., 2006). In contrast, *in vitro*, rat POMC 1-28 stimulated proliferation in cultured rat glomerulosa and fasciculata/reticularis cells, but ACTH was inhibitory (Mattos et al., 2011). The discrepancy between the stimulatory actions of ACTH on adrenal cell proliferation *in vivo* and its inhibitory actions *in vitro* is difficult to resolve. It is possible that other tissue components, such as the vasculature (Figure 4), absent from cell cultures, are required for the proliferative actions of ACTH *in vivo* (Thomas et al., 2004).

Although dependence on ACTH is paramount, other factors are known to modulate fasciculata cell secretory activity, including cytokines, which are differentially produced throughout the cortex, and have specific actions on individual cell types (Spangelo et al., 1995; Judd, 1998; Koldzic-Zivanovic et al., 2006; Woods and Judd, 2008), and neurotransmitters (Ehrhart-Bornstein et al., 2000). The possibility of medullary, neural and vascular regulation of cortical function is emphasized by the intimate relationship between these adrenal gland components (Figure 4).

### Regulation of the reticularis

The control of adrenal androgen secretion is problematic, in that although supported by ACTH, secretion of androstenedione and DHEA is dissociated from cortisol under some conditions, and particularly during adrenarche which has led some authors to postulate additional pituitary or other factors (McKenna et al., 1997; l'Allemand and Biason-Lauber, 2000), and, more recently, melatonin was found to stimulate DHEA release from cultured adrenals of the solitary hamster *Phodopus sungorus* (Rendon et al., 2015). However, the factors causing the increase of adrenal androgen secretion during adrenarche are largely unknown, as are those causing the later decline, or adrenopause (Dharia and Parker, 2004; Bird, 2012) leading some authors to postulate the importance of intra-adrenal factors; these could include growth factors (l'Allemand and Biason-Lauber, 2000), cytokines or even steroids. Cortisol stimulates DHEA secretion in H295R cells by inhibiting 3β-HSD (Topor et al., 2011), DHEA modulates chromaffin cell proliferation and differentiation (Chung K. F. et al., 2011) and LH receptors are present in human fasciculata and reticularis, albeit at a low level (Pabon et al., 1996; Rao, 2010). However, another provocative finding in this regard is that inhibition of seladin-1, which is strongly expressed in the fasciculata and reticularis of the human adrenal, specifically inhibits the ACTH induced secretion of DHEA in human adrenal cells, while having no effect on cortisol output (Battista et al., 2009). This awaits physiological interpretation.

## The significance of adrenocortical zonation—how does the standard model stand up?

That adrenocortical cells arise in the capsular or subcapsular region of the gland and migrate inwards is now virtually unchallenged. That proliferation can occur throughout the gland is also clear, but does not affect the main thesis, even given there appear to be exceptional situations in which migration may be reversed.

It is also clear that the distribution of enzymes and other components between the zones show sharp phenotypic differences between the glomerulosa and the fasciculata, and slightly less sharp differences between the fasciculata and the reticularis, hence the inwardly migrating cells undergo more or less profound phenotypic changes in their lifespan. What remains unclear is how this is achieved, although recent advances in our understanding of the role of morphogens such as wnt and shh give some powerful guides: these go a long way to explaining how the glomerulosa becomes differentiated from the capsule. Remaining to be determined is how the fasciculata becomes distinct from the glomerulosa, or the reticularis from the fasciculata. What morphogens are involved here?

As well as the speculation that they could conceivably be products of neural or vascular components of the gland (Figure 4), one school of thought holds that it could be the steroid products themselves. From *in vitro* studies, and on the basis that there may be a gradient of steroids from the outer to the inner part of the cortex, Hornsby and colleagues postulated that this results in high concentrations of steroids in the inner zones, which, by acting as pseudosubstrates, could directly compromise cytochrome P450 structure and function, in this way perhaps inhibiting hydroxylase activity in the reticularis, for example (Hornsby, 1987). Though this is contrary to the usual concept of morphogen diffusion which posits a decrease in concentration with distance from the source, it acquires new life, and a new mechanism, with the characterization of GR in the human adrenal cortex, particularly in the reticularis (Paust et al., 2006).

That the individual zones have defined steroidogenic roles is clear. Whether they are completely autonomous is less so. Rather, because of the paucity of other steroidogenic enzymes in the CYP11B2 expressing cells of the glomerulosa, it is arguable that the substrate for CYP11B2 must come from the fasciculata, and the different ways this could occur are via cell-cell contact, or by uptake from the general circulation, as presumably is the case for extra-adrenal CYP11B2 thought by some to be expressed in the vasculature and other tissues (Hatakeyama et al., 1996; Taves et al., 2011; though not by others, MacKenzie et al., 2012) or by diffusion. Similarly, the production of androstenedione by the human gland would seem, on the basis of the zonal distribution of enzymes possibly to be a reflection of the availability of DHEA from the reticularis to 3β-HSD in the fasciculata.

If that is the case, then the regulation of these secretory products must also reflect co-operation between zones. Given the distribution of mRNA coding for MC2R in the rat adrenal, in the inner ZU, and fasciculata/reticularis but not in the CYP11B2 region of the glomerulosa (Gorrigan et al., 2011), the well-recognized stimulation of aldosterone secretion by ACTH is otherwise difficult to explain. Of course it is still possible that it is the MC5R in the glomerulosa to which ACTH binds. The actions of angiotensin II and α-MSH on aldosterone secretion in the rat are equally difficult to understand, because the appropriate receptors, AT1R and MC5R respectively, do not appear to coincide with CYP11A1, thus cholesterol side chain cleavage, the recognized site in the pathway for stimulation of steroid hormone secretion, seemingly cannot be directly affected by these agents (Figure 3).

Taken together, all this evidence seems to suggest it may be mistaken to think of the adrenal cortex, or even the whole adrenal gland, as simply a collection of different types of cells, each independently performing its role in isolation. Rather we should think of these different cell types as acting in concert. We have understood for some time now that there are intraadrenal mechanisms that modify and refine the signals from the systemic regulators. These comprise medullary products, cytokines, the adrenal renal-angiotensin system, among others, and have been reviewed elsewhere (Ehrhart-Bornstein et al., 1998). Extending this has been conceptually problematic in view of the apparently confounding effect of the blood flow through the gland, which would seemingly limit the action any cell might have on other than its closest neighbors by secreted products.

We now know that secreted products from adrenocortical cells can indeed pervade the gland through diffusion, and this has been demonstrated by the wnt-catenin and shh-Gli signaling systems. So if it is possible for these products then there is no reason why it cannot be the case for others, including perhaps catecholamines, cytokines, and the steroids themselves. This may give a partial explanation for the architecture of the gland, one of the problems posed at the start of this article—why are the zones arranged as concentric spheres? One answer may be that this has the potential to maximize the likelihood of diffused products arriving at their targets. If we take as an example the secretion of shh from its origin in the ZU, random walk diffusion to its target in the inner capsule is better achieved when the capsule completely envelops the glomerulosa than it would be if the system were more open, or composed of sheets, like a mesentery or secretory epithelium.

In addition, there is absolutely no reason to suppose the steroids themselves do not move between the zones, as substrates or modifiers. How else can we explain how aldosterone or androstenedione are synthesized? And, given that MC2R and CYP11A1 are expressed in the ZU and fasciculata, but not the CYP11B2 expressing cells of the rat glomerulosa, how else can we explain how ACTH has its undoubted action on aldosterone secretion, other than by the enhanced provision of steroid substrates from the ZU or the fasciculata to the CYP11B2 cells in the glomerulosa?

Like the human body itself, the function of the adrenal gland is an integrated whole, much greater than the sum of its parts. We should aim to think of it that way.

## Author contributions

GV researched and wrote this article.

### Conflict of interest statement

The author declares that the research was conducted in the absence of any commercial or financial relationships that could be construed as a potential conflict of interest.
